# Energy Performance Analysis and Modelling of LoRa Prototyping Boards

**DOI:** 10.3390/s21237992

**Published:** 2021-11-30

**Authors:** Solomon Ould, Nick S. Bennett

**Affiliations:** 1 Centre for Advanced Manufacturing, University of Technology Sydney, Broadway, Sydney, NSW 2007, Australia; nicholas.bennett@uts.edu.au; 2 Radio Frequency and Communication Technologies (RFCT) Lab, University of Technology Sydney, Broadway, Sydney, NSW 2007, Australia

**Keywords:** LoRa, energy performance, LoRaWAN, IIOT, wireless networking

## Abstract

LoRaWAN has gained significant attention for Internet-of-Things (IOT) applications due to its low power consumption and long range potential for data transmission. While there is a significant body of work assessing LoRA coverage and data transmission characteristics, there is a lack of data available about commercially available LoRa prototyping boards and their power consumption, in relation to their features. It is currently difficult to estimate the power consumption of a LoRa module operating under different transmission profiles, due to a lack of manufacturer data available. In this study, power testing has been carried out on physical hardware and significant variation was found in the power consumption of competing boards, all marketed as “extremely low power”. In this paper, testing results are presented alongside an experimentally-derived power model for the lowest power LoRa module, and power requirements are compared to firmware settings. The power analysis adds to existing work showing trends in data-rate and transmission power settings effects on electrical power consumption. The model’s accuracy is experimentally verified and shows acceptable agreement to estimated values. Finally, applications for the model are presented by way of a hypothetical scenario and calculations performed in order to estimate battery life and energy consumption for varying data transmission intervals.

## 1. Introduction

LoRa has several unique characteristics in the Low Power Wide Area Network (LPWAN) space, but its principal advantages are long range communication with extremely low power draw [1]. LoRa is based on Chirp Spread Spectrum (CSS) radio modulation in the 433, 868 and 915 MHz bands [2]. LoRa technology brings the potential for battery-powered sensors to operate for up to 10 years without requiring recharging or battery replacement [3]. This provides multiple new applications for Internet-of-Things (IOT) devices covering large distances and requiring minimal infrastructure expenditure. A single LoRa gateway is able to support thousands of concurrent devices [4] which provides the potential for sensor networks to be rapidly deployed over expansive sites cheaply, and quickly integrated into existing systems [5]. In order to accomplish LoRaWAN networks over large distances, a star of stars network topology is typically utilised with several gateways [6]. Recently simulation has been completed showing that LoRa with appropriate modulation improvements can be used for reliable satelite communications in low earth orbit applications [7].

LoRa as a technology is placed inside the LPWAN space with other notable technologies such as Bluetooth Low Energy, Sigfox, NB-IoT, Ingenu, EC-GSM, to name a few. Within this category technologies are often grouped based on their use of a licensed or unlicensed frequency band, with Sigfox, LoRa, and Ingenu using unlicensed frequencies [8]. The focus of this work is long-range battery-powered nodes, which excludes short range technologies such as Bluetooth. Table 1 shows a high level comparison of the most common wireless networking protocols and LoRa. Another technology, (not shown in Table 1) is Sigfox, both LoRa and Sigfox offer high range low power communication, however Sigfox does not allow for private networks and gateways, is limited to a 12 Byte message size, and has restrictions on the number of messages sent per day [9]. LoRa has a larger maximum payload size and no restriction on message size. LoRa is well suited for our use case, allowing long-range data transmission, encryption, and the ability to leverage private gateways and be used in an offline installation. In the last 10 years, LoRa has garnered an exponential increase in interest due to its properties including low power, long-range, and low-cost chipset [10].

In order for the benefits of LoRa to be properly utilised for a remote node, the power requirements are key. In many application environments it may not be necessary to transmit data using the maximum distance settings. For example Liang et al. showed that a single LoRa gateway could maintain acceptable signal strength placed at any position in a 12 story building [12]. In this case, sensors destined to be closer to the gateway could operate at lower transmission power and higher data-rates to save power. Most devices are designed to be powered through battery, or energy harvesting devices (e.g., small solar panels), where every mW of power consumed to send a message is important to the usability of the device. If power consumption per message is brought to an absolute minimum then either the battery life of the unit can be extended or the transmission interval can be increased in energy harvesting applications.

In order to prototype applications for this technology it is commonplace to begin by purchasing a development kit containing a Micro Controller Unit (MCU) and a LoRa radio. There are many such prototype boards available and those which we interrogated are marketed using the power usage based on deep sleep consumption. This value is incredibly low with typical MCU units able to remain asleep with current values measured in the μA range. However, almost no power consumption figures are presented for transmission in marketing material. Given the lack of manufacturer data available on transmission power requirements it is thus impossible to to make an estimate of the power required for a specific use case. This initial estimate is crucial to investigate as it can provide a fast way to validate the likelihood of success for an application of the technology, i.e., if a specific size, power input requirement, and message interval is specified an engineer is unable to estimate if a LoRa module can successfully provide a solution.

With the work presented in this paper a formulaic power estimate is provided allowing a designer to consider the likely transmission characteristics required and subsequently estimate the battery size required and transmission interval possible.

## 2. Methodology

We first compared energy performance of multiple boards and selected the lowest power board for in depth analysis and modelling.

### 2.1. Equipment

Boards were selected due to prevalence in the market place, and boards marketed as “low power consumption” were preferred, availability with current supply chain issues was also an influence. The boards chosen are displayed in Table 2.

All boards were tested using default parameters for the channel plan selected (EU868) [6] the parameters used during testing are listed in Table 3. The independent variables selected for testing were the data rate, TX power, and payload size.

The cost of the boards was not considered as a critical factor for the experiment given the primary aim to ascertain the lowest power settings possible to achieve transmission using commercial off-the-shelf components. However, all boards where available for purchase for less than USD 50 at the time of writing. The price of the boards varied based on the features available and processing power of the MCU. It can be seen from Table 2 that the most expensive board is the LoPy4 which contains a long list of advanced features including a Python micro environment, and integrated radios to enable communication over WIFI, Sigfox, BLE, and LoRa [13]. In the context of our experiments, these features are not required as we are focusing solely on the LoRa radio aspect.

The chosen power testing apparatus was the Keithley 2450 SourceMeter with specifications shown in Table 4.

### 2.2. Method

In order to initially compare the power requirements for the different boards, firmware was flashed onto each board using the same parameter values. The test program was designed to be as simple as possible, it comprised establishing a link to the gateway using data rate (DR) 0 and TX power 12, followed by a 30 s delay, and finally sending a 2 Byte message. All boards sent the same message and contained the same delay and transmission settings. The 30 s delay was added in order to differentiate the join and transmission events in post analysis. Another consideration in the board comparison was consistency, the test was run 5 times on each board with the variability between the runs analysed.When the lowest power consumption board was identified, a further series of tests was performed in order to analyze the effects of message size, TX power setting, and data rate, on the power consumption of that board.

Using the information contained in the testing, a model was derived using polynomial fitting in order to predict the energy consumption of the board under various transmission settings. Finally, the models accuracy was analysed through comparing actual power consumption figures with those estimated. The testing was time consuming, as it required re-flashing new firmware to the board before any changes were made, resulting in a slow iteration time in order to reveal the trends in power consumption for these control parameters.

## 3. Multiple Boards Comparison

Background research into the factors which effect the power consumption of a LoRa device result in an extremely long list of factors. Testing the impact of each factor individually would be prohibitively slow, so, where possible, all factors were fixed in order to attempt to standardise the testing environment between the boards. The antenna, power supply, firmware settings, gateway, application server, distance from gateway, message contents, delay, and room temperature, all remained constant.

**Figure 1:**
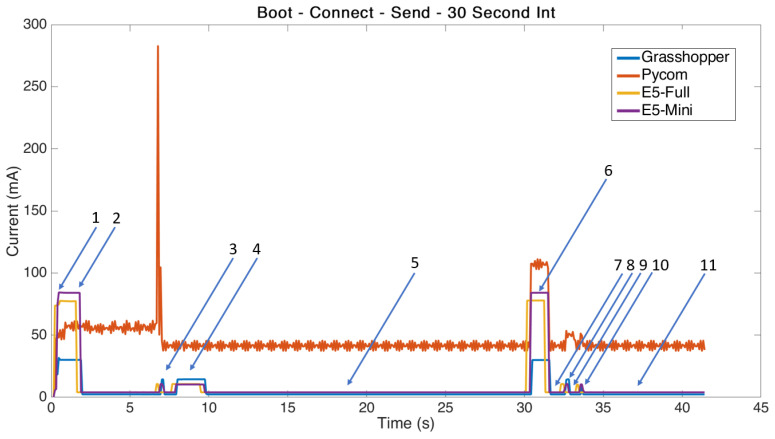
Power consumption for different prototyping boards. (state descriptions in Table 5).

The power consumption data were recorded using the SourceMeter in CSV export function at 100 Rdgs/S and the values measured were current and source voltage. The results were analysed in Matlab and the resulting current curves synchronised to provide the outcome plot show in Figure 1.

From analysis of Figure 1 we can see some interesting behavior. Firstly, it is quickly apparent that the Pycom board uses significantly more power than that of the others when performing the same task. It should be noted that the Pycom board contains a Python micro environment and has far more advanced features than that of the other boards tested. However, it is also marketed as a low power consumption device and it is clearly evident that its idle power consumption is significantly higher than that of the more simple boards. This follows logically as any device with higher computing performance will consume more power and generate more heat in general. However, in applications such as remote battery powered sensors it is unlikely that the extra computing performance of the Pycom board is necessary given the significant power cost attributed to it.

**Figure 2:**
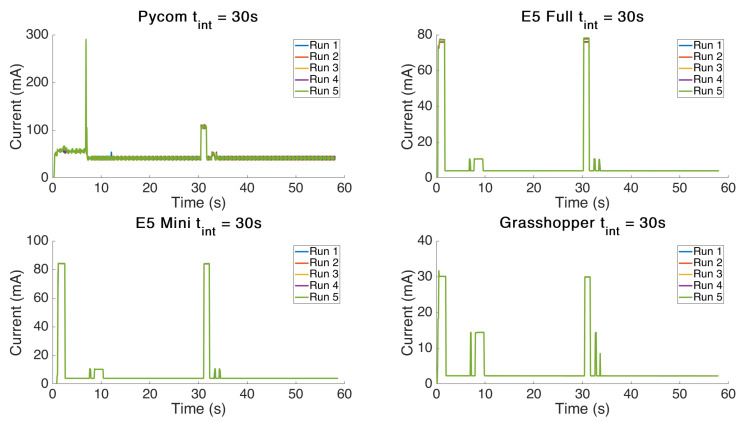
Multiple board power variability over multiple runs.

In order to analyse how repeatable the power consumption required for the test was, repeats were carried out and the plots for this test are shown in Figure 2. It can be concluded that multiple tests show very little variance in the power profile consumed to accomplish the same task and there is very little variability. However, the Pycom board showed greater variability with a standard deviation of 0.14 W·s as compared to an average deviation of 0.004 W·s for the other boards.

**Table 5:** Identified Power Profile Stages and Numerical Results.

State Number	Description	E5-Full	E5-Full	E5-Mini	E5-Mini	Pycom	Pycom	Grhppr	Grhppr
Time (ms)	I (mA)	Time (ms)	I (mA)	Time (ms)	I (mA)	Time (ms)	I (mA)		
1	Wake Up	483	74.7	483	84.4	896	51.9	483	31.7
2	Join Transmission	1070	77.2	1324	84.1	6368	55.0	1406	30.1
3	Join Receive 1	248	10.6	331	10.7	165	282.8	248	14.4
4	Join Receive 2	1902	10.7	1986	14.48	166	104.7	1986	14.5
5	Programmed Wait	-	-	-	-	-	-	-	-
6	Transmission	1080	77.9	1080	84.1	1320	111.2	1240	30.0
7	Wait First Window	910	4.0	910	4.0	910	45.6	910	2.3
8	Receive First Window	330	10.6	330	10.7	420	53.5	330	14.5
9	Wait Second Window	660	4.0	660	4.0	410	33.4	660	2.3
10	Receive Second Window	250	10.2	330	10.4	250	51.3	170	8.5
11	Idle	-	-	-	-	-	-	-	-

Casals et al. found that there are 11 distinct states in the LoRa modem transmission power profile and these are described in detail in their paper [15]. For comparison purposes their states are displayed in Table 6. The results from our power profile analysis, shown in Figure 1 and Figure 2, show agreement with the majority of the phases described in their work; however, there were some discrepancies. Casals et al.’s power profile does not appear to capture a difference between the “joining” of the network and transmission of the message. This may be because they did not program an intentional delay as we did, which may have resulted in the joining and message transmittal events occurring at the same time, or perhaps their results were from a board which had already joined the network and had been powered on for some time. The power profiles we obtained clearly show a spike approximately 5 s after boot up, which is consistent in timing with the network acknowledgement of joining. We then observe the programmed delay followed by transmission. It is likely this separate power profile step was amalgamated into their analysis due to immediately transmitting the data after joining. This is interesting to note as the current profile is different for both the joining and normal message transmission events.

By interrogating our results shown in Table 5, it is evident that the timing is quite consistent for the duration of events identified. The Pycom board’s results show slightly different timing duration for events and this is likely due to error caused by the noise in the generated current waveform. The noise present on the generated data made it more difficult to accurately identify when different stages are beginning and thus affects the accuracy of the timing results for this board.

It was expected that timing would be consistent due to the nature of the LoRa protocol determining when each event takes place. The duration for the first and second receive windows are detailed in the LoRaWAN regional parameters document. There is critical difference between the first and second receive window specifications such that the first window allows a flexible data rate (DR) to be adjusted by the user with the second receive window being fixed at a default frequency of 869.525 MHz [15]. For this reason, it is likely that there would be some variation in receive window power consumption for different data rate up-links depending on which window received the transmission.

In order to enable message receiving, the window timing is not static and instead relies on a fixed duration to ascertain if a message preamble has been detected [15]. If a preamble is detected then the modem remains active for the duration of time required to receive a complete message. This principle can be clearly in Figure 1 and Figure 2 if we look at the duration of time consumed by “Join Receive 2” in contrast to all other receive windows. The program used does not receive any data from the LoRa gateway during normal message transmittal but during the “joining” event it must receive a message to indicate the success or failure of that event, which results in the significantly longer window time of this state in our testing.

The Pycom board proved difficult to analyse during the “Join Event”, showing a completely different waveform and not returning to idle while waiting for a confirmation of join. This is potentially caused by the board containing a different modem and receiving the confirmation during the first receive window. The Pycom board showed an extremely large spike at the expected time of the first receive, which appears to be a transmission, so we were unable to identify the states accurately for this board during this period.

It was unexpected that the power consumption for events would have such a large variability between the boards. With the principal difference in these boards being the MCU controlling the LoRa modem some variation was inevitable; however, we are seeing differences in the magnitude of 4 times the power required during transmission events. The MCU power consumption difference in theory should mostly affect the idle, sleep, and computation phases with a relatively static power offset due to the different clock speeds during transmission. Indeed we saw behaviour similar to this between the E5-Full and E5-Mini boards. The large variance overall in testing is difficult to rationalise given that all parameters in the software remained the same and the same antenna and gateway were used. The difference likely lies in the modem design and firmware application for each board.

Ultimately, these are significant differences which will affect the eventual end product design considerably, and are based on best case scenarios for power consumption, i.e., there are no calculations taking place on the boards or sensors attached, we are only sending raw values.

**Figure 3:**
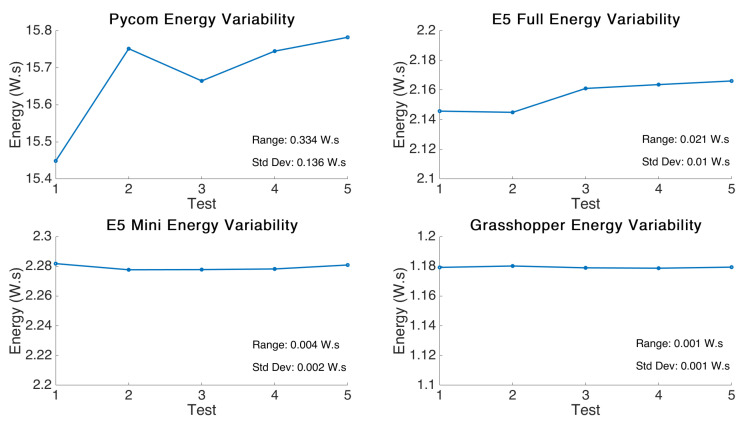
Multiple board total energy variability over multiple runs (calculated using individual live sample voltage).

The variability between different test runs of the same firmware was found to be very low. Figure 3 shows the variance between the 5 tests that were performed on each board and illustrates the very low standard deviation and range between data points.

### Comparison Summary

From research carried out at the outset of this work, a long list of factors influencing the power of our devices was created. Testing the effects of each of them would be prohibitively slow, so we fixed the factors which were appropriate, and tested for parameters we defined as most relevant.

The software parameters which were shown in literature to have the most significant impact on power consumption were the data rate setting, the transmission power settings and the message size. The transmission interval obviously has a significant impact on the device power consumption as well, but this is adjustable to the users requirement and varying the interval will change the overall device power consumption but has no impact on the single message power consumption.

The key insights from the multiple board testing experiment were that the power required varies depending on the construction of the board. This is logical as each board uses a different MCU and thus has a different clock rate. However, considering each board is marketed as low power and manufacturers do not actually provide any power consumption data other than the minimum rated power supply it is clearly important to actually test these devices, as one could falsely believe they are each equivalent. The low variability in power used between tests was encouraging, this shows that if an appropriate model is developed we can accurately determine the power required to send a single message and then multiply this by the number of messages per day and factor in the devices sleep current to estimate the total power consumption.

## 4. Grasshopper Board In-Depth Analysis

The multiple board comparison testing showed that for the single message experiment the Grasshopper board had the lowest total overall power consumption in every test performed using the same settings and environment. For this reason, this board was selected for further analysis and testing in order to generate a model.

We performed a series of tests on this board by fixing all parameters to the defaults shown in Table 3 and then varied one parameter of interest in sequence, in order to understand the effects on power consumption. These tests were time consuming due to the requirement to flash new firmware to the board before each run by placing the device in USB boot load mode and then compiling and uploading the firmware. Once the new firmware was in place the device was removed from the computer and installed on the test bench connected to the SourceMeter. Several runs of each parameter were interrogated to ensure consistent results for the average power consumption and the online gateway was checked to ensure the message was successfully delivered. At the end of this process, we had a single data point for the power curve at that setting and the process was repeated using multiple settings across the entire range of acceptable values. In some cases, we intentionally input values outside the allowable parameters to investigate the behaviour of the device with too large or small values configured for settings.

The results from these experiments are compiled into a series of plots shown in the following pages and are broken down into the results of:

- Multiple Frame Power Analysis
- Message Size Comparison
- TX Power Comparison, and
- Data Rate Comparison.

These tests are and their results, are described in detail in the following sub sections.

### 4.1. Multiple Frame Analysis

**Figure 4:**
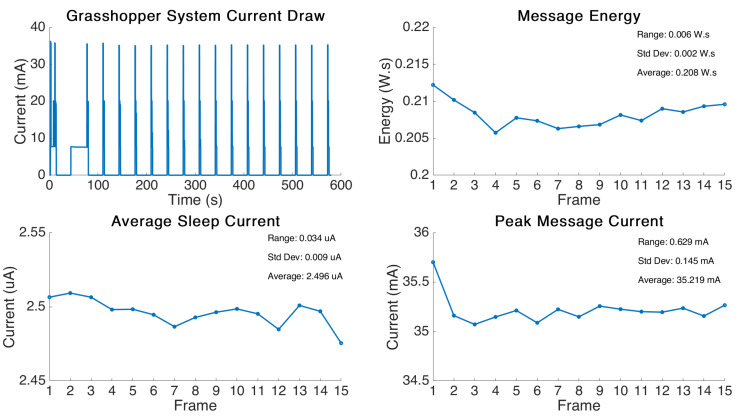
Grasshopper board multiple frame power analysis $t_{int}$ = 30 s.

The graphical results from the test are displayed in Figure 4. This test was performed slightly different to the one earlier in the Multiple Boards Comparison section. The Grasshopper board was programmed to send a consistent 2 Byte message at an interval of 30 s. The period in between the messages was this time configured to be “deep sleep” state. This allowed us to analyse the sleeping current required in this state in the period in between message transmittals. The results were then post processed in Matlab to find the message power variance, peak message current, and average sleep current.

The test showed that similarly to the previous test, variability was very low with a range of only 6 mWs per message and a standard deviation of 2 mWs. The average sleep current was determined to be 2.50 μA. This result for sleep current fits extremely well with the advertised deep sleep current of 2–3 μA.

The peak message current was measured to be 35.2 mA on average with a similarly low variance comprised of a range of 0.63 mA and a standard deviation of 0.15 mA.

An interesting takeaway from this experiment was that the first two messages show higher power consumption. In order to ensure this was not an outlier, the test was performed several times and the results for the first two frames remained the same. This behaviour appears to be due to the timing being influenced by how long the device takes to join the network in the first frame. During the first frame transmission, the device attempts to connect to the network first, then immediately sends a frame, then sleeps for 30 s. However, it appears the modem does not allow a re-transmision straight away after wake-up and instead causes the device to wait to send the message. This causes the higher power consumption while the device constantly checks for the ‘modem busy’ flag and then eventually sends the second message. This behaviour disappears after the third message and the power profile is consistent thereafter. This behaviour was intriguing and we were able to replicate it by deliberately setting the transmission interval too high, for example to 10 s. The device would similarly receive the ‘modem busy’ flag and would wait, using high power, while constantly checking for the modem to be available to send. This behaviour disappeared when higher transmission intervals were employed.

The results from this test provided the required data for the deep sleep, which will be incorporated in power estimation for the device in this state in the model, and also provide further evidence of the extremely low variability of these devices when sending messages with a fixed parameter set.

### 4.2. Data Rate Analysis

**Figure 5:**
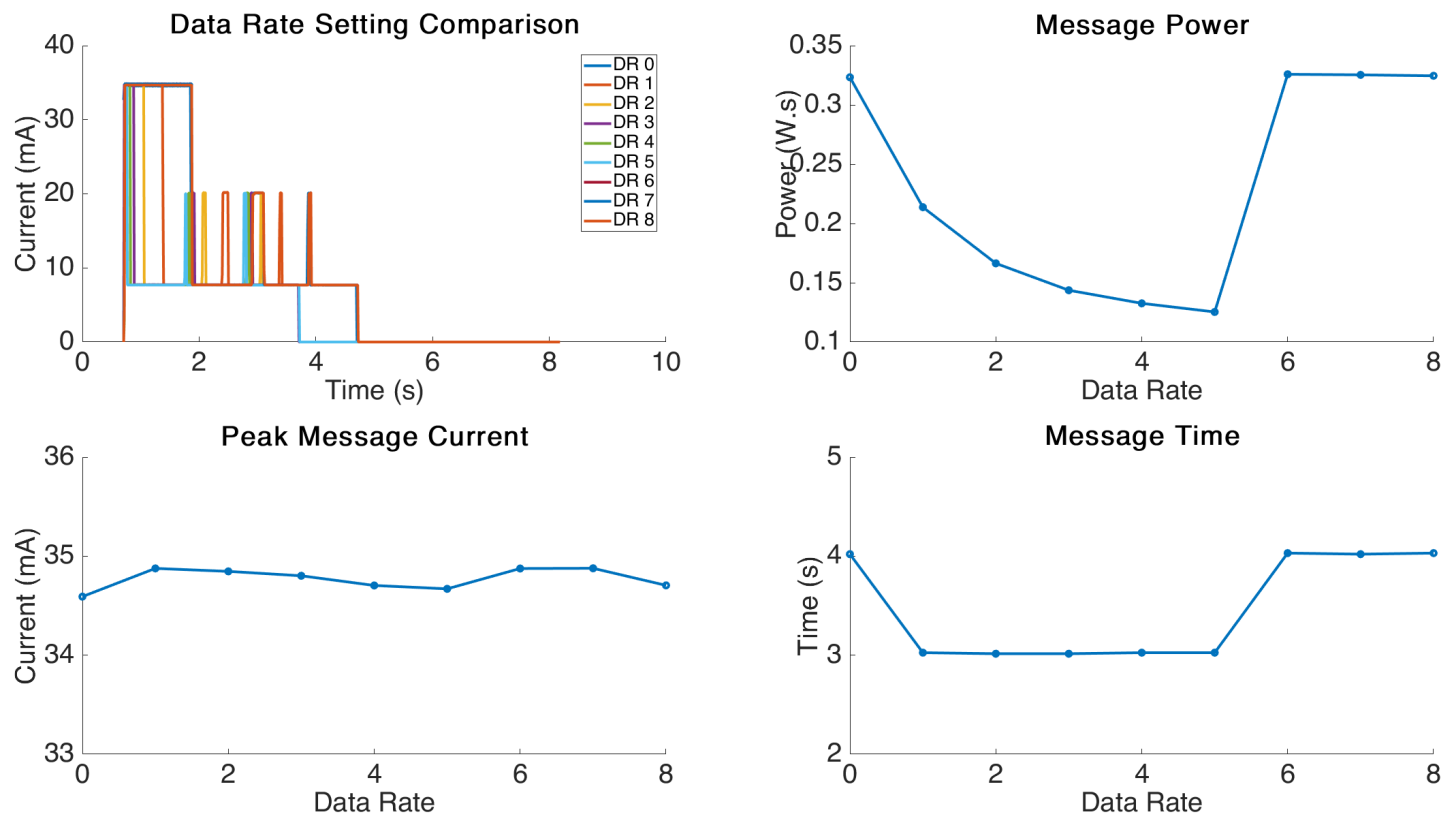
Data Rate setting comparison.

The graphical results for the data rate testing are displayed in Figure 5. This experiment was performed by sending a standard 2 Byte message using a transmission interval of 30 s and varying the data rate setting from DR-0 to DR-8. The system is unable to deliver at data rate 8 as this is an invalid setting; however, we wanted to investigate the behaviour with an erroneous data rate input. The data rate setting corresponds to an adjustment of the bit rate for transmission which is set in the range 300 bit/s to 5.5 kbit/s [16]. Increasing the bit rate of the transmission will in turn reduce the time on air required for the message and thus should reduce power consumption, but will also limit the range [16].

Looking at the power profile delivered from the tests we can see some interesting behaviour. The message power required ultimately reduces with valid increases in the DR setting. This shows good agreement with the results published in other works on this topic [15,16,17]. We can further see that as the transmission bit rate increases there is a staged reduction in message delivery timing from approximately 4 s down to 3 s. This decrease in message time is not linear and instead only steps down to a faster message time at DR1 and then stays static at the 4 s mark. These are likely agreed timings set in the LoRa protocol.

When the data rate is set to an erroneous value (i.e., >DR5) the firmware clearly resorts to using DR0 as the timing and message power immediately returns to match these settings.

The clear trend visible with the data rate setting is that increasing the data rate to the highest setting possible with the transmission distance required will result in an exponential decline in overall power consumption for the device; however, after DR1, it will have no impact on the message delivery time.

### 4.3. Message Size Analysis

**Figure 6:**
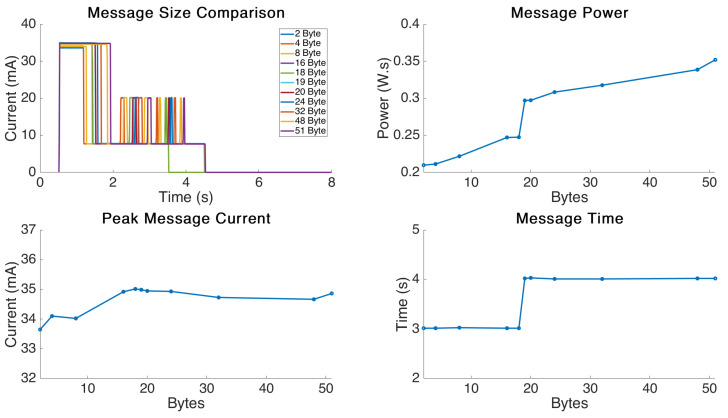
Message size comparison.

The graphical results for the message size testing are displayed in Figure 6. This experiment was performed by fixing the static values to defaults similar to the previous experiments and slowly increasing the message size in order to generate a trend line for the effect of message size on power consumption.

The most interesting behaviour shown in the experiment was the stepped message time result. This result matches previous results such as in the data rate experiment which resulted in a similar stepped response for message delivery time rather than a linear increase. It appears the LoRa protocol opens a further window for larger messages which results in it remaining open for a fixed duration regardless of when the message is completed. We found this behaviour to begin at a message size of 19 Bytes and continue right through until the maximum size we tested which was 51 Bytes, the maximum for DR0 [6].

The peak message current showed some variation but there was no obvious trend and it appeared random, the variation was less than 5 mA between highest and lowest readings.

The message power showed a linear increase with message size up to the threshold value of 19 Bytes and then a significant jump caused by the opening of the longer transmission window. It then showed a similar linear increase from 19 Bytes to the maximum tested value of 51 Bytes.

These results are interesting as they show a significant power ‘overhead’ for increasing the data transmitted over this threshold by one Byte. This information is extremely important for design engineers who may be able to tweak designs in order to remain under this threshold value and gain significant energy savings resulting in longer battery life.

The message size analysis shows important threshold behaviour which engineers should be aware of if trying to optimize hardware for extended battery life or higher transmission intervals. With battery-powered IOT hardware it is logical to reduce the message size where ever possible, but this insight shows the dramatic effect a single extra byte can have on of the overall power consumption.

### 4.4. TX Power Analysis

**Figure 7:**
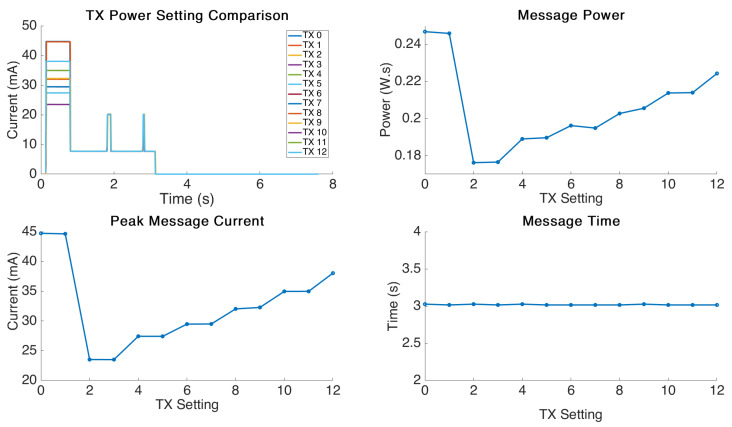
TX power comparison.

The graphical results for the message size testing are displayed in Figure 7. This experiment was performed using the default values of DR-0 and a 2 Byte transmission message. The TX power setting was then increased linearly from setting 0 to setting 12. It may be tempting to assume that one will always need maximum TX power for an application, however as compared to Bluetooth and WiFi, LoRa signal transmissions can reach up to kilometers with line of sight [18]. For this reason, there are actually many applications in which we could make an estimation of the TX power required by using a fraction of the theoretical maximum based on the application environment. Gloria et al. showed that you can achieve up to a 48% reduction in power usage by adapting the TX power of the end node to different situations and this can be accomplished dynamically [19].

As expected in this section, we see a relatively stable linear power increase with each TX power step as a trend. We also see, as expected, no effect on the message delivery time as a result of manipulating this setting.

We see interesting behaviour at TX setting 1 which causes the highest power consumption possible and appears to be caused by a firmware related problem indicating that TX power setting 2 is actually the lowest permissible value. The documentation for this board was not provided or discoverable, so we were unable to determine the maximum and minimum permitted settings. It appears that the firmware compilation, when faced with an illegal setting, defaults to maximum power output and causes a large jump in power consumption as a result.

Using the data obtained for the linear increase in power consumption, we are able to estimate the impact on the overall consumption from this attribute, which is covered next in the modelling section.

## 5. Power Model

With the data we collected in the previous sections, we were able to produce a model using linear regression, and fit polynomials to predict the estimated contribution factor from each firmware setting. Due to the natural stepping, which occurred in the message size, the model was separated based on the input as being less than 19 Bytes or greater than 19 Bytes. In order for the reader to replicate the estimation program if required, we have provided the output polynomial coefficients from the regression below:

Datarate Power Contribution (0–5)

$E_{dr}=\left(p1\times x^{4}+p2\times x^{3}+p3\times x^{2}+p4\times x+p5\right)-0.208$
p1=0.00061847
p2=−0.0092522
p3=0.053733
p4=−0.15423
p5=0.32323

TX Value Power Contribution (2–12)

$E_{tx}=\left(p1\times x+p2\right)-0.208$
p1=0.0045733
p2=0.1664

Message Size Power Contribution (1–18 Bytes)

$E_{size}=\left(p1\times x+p2\right)-0.208$
p1=0.0025994
p2=0.20252

Message Size Power Contribution (18–51 Bytes)

$E_{size}=\left(p1\times x+p2\right)-0.208$
p1=0.0015828
p2=0.26733

Total Power if DR < 2

$E_{total}=0.208+E_{dr}+E_{tx}+E_{size}$

Total Power if DR > 2

$E_{total}=0.208+E_{dr}+E_{tx}$

The above formulas yield a variable output power estimate measured in W·s for the amount of energy required to send a single frame of the specified size at the specified data rate and transmission power.

### 5.1. Power Model Verification

Once the model had been generated based on the testing results, we verified its accuracy using further testing. Input parameters spread over the ranges were randomly selected and and an estimate recorded. The parameters were then compiled and uploaded to the boards and each test point was compared to the experimentally recorded measurement.

Initial testing of our first model iteration identified peaks were the model lacked accuracy and these occurred systemically where there was a high data rate and high message size. We became aware that at low data rates the message size had a significant impact on power consumption, but this effect was greatly diminished at data rates higher than setting 2. We hypothesise that due to the increased bit rate at these data rates, the effect of the larger message was negligible on the overall consumption and updated the model to contain the ‘if’ statement. That is, in the case of a data rate of less than 2, the estimated power includes the message size contribution, but for any other data rate it is ignored.

**Figure 8:**
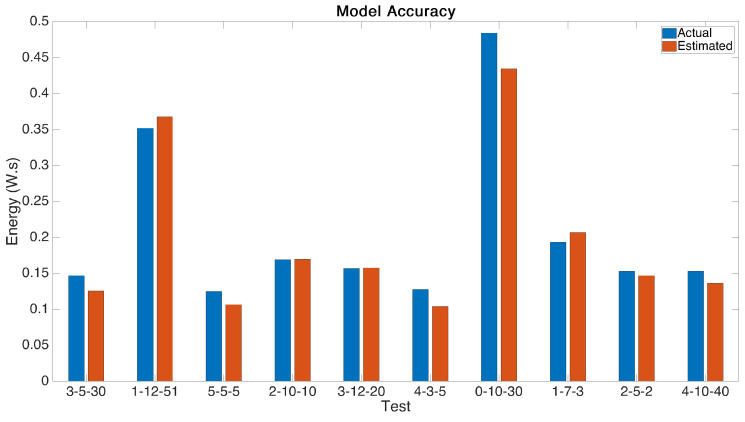
Model verification, comparing actual, measured experimental energy with that predicted by the model.

With this change we were able to arrive at the results shown in Figure 8. The results show an average mean variation between measured and expected power of 9%.

### 5.2. Sensors

The testing that has been performed on the Grasshopper board thus far has accounted for variations in message size, data rate, and TX power. With the model created we are able to accurately estimate the power required at different transmission intervals to transmit a message. However, we are currently unable to estimate the power that will be taken by the sensors, which of course read the value that is to be sent.

Several preliminary measurements were carried out using a range of common sensors in order to estimate the power used by them during operation. The sensors did not experience any dynamic power variation and are easily modeled with a static power draw figure over time. The sensors we tested are shown in Table 7.

**Figure 9:**
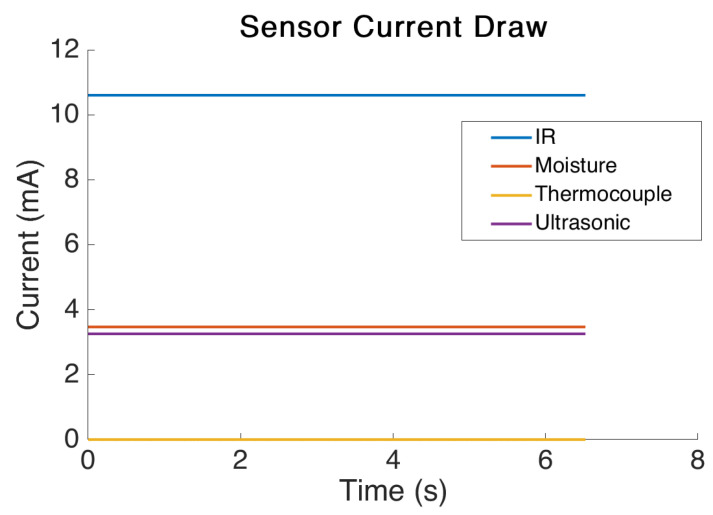
Sensor current draw test results.

The static power draw test results are shown in Figure 9. From the results it is clear that the sensors use an extremely small amount of power. The IR sensor used the most power with an average power draw of 10.6 mA. The lowest power sensor tested was the digital thermocouple which used only 0.5 μA of current to power the onboard chip. This figure is the power draw during operation and each sensor utilised must be considered for how long it actually requires being operated. For example, there is no necessity to power a moisture sensor after a reading has taken place but there may be a minimum amount of time it must be powered before an accurate reading can be obtained.

These variable timing figures will need to be investigated and considered for any potential design use case and falls outside the scope of this paper. For the purposes of simple power estimation we can apply an additional sensor current draw ‘overhead’ for the duration of time the LoRa board is running as a worst case. It should be noted there are some sensors (for example CO_2_ gas sensors) which require running for several minutes before a reading can be taken and this would dramatically effect the overall power consumption, so would need to be considered differently.

### 5.3. Applying the Model

With the data generated up to this point and the model presented, we can now perform an application on a hypothetical situation. We define a use case as a digital sensor which is approximately 1/4 of the maximum transmission distance from the gateway, which will be sending a single long variable sensor output. We then select likely model settings of DR2, TX3, and a message size of 2 Bytes.

The previously presented model provides an estimated single frame energy of:

$E_{frame}=0.1377$ W·s

We then define the transmission interval in seconds as:

$t_{int}=30$ s

With these figures we can estimate the energy consumption per day as a fraction of the time spent in the message delivery mode, and sleep mode at the given interval. We noted previously that frame time is split dependant upon data rate and message size, from previous results; in this case, it will be 3 s:

$t_{frame}=3$ s

Therefore:

$E_{sleep}=I_{sleep}\;V_{ref}\;\left(86,400-\frac{86,400\;t_{message}}{t_{int}}\right)$

$E_{message}=\frac{86,400}{t_{int}}E_{frame}$,

and finally the daily power requirement will be:

$E_{total}=E_{sleep}+E_{message}$

If we then assign a battery to the device, we can calculate the estimated battery life between recharge:

$t_{days}=\frac{E_{battery}}{E_{total}}$

This method is not designed to completely remove the requirement for testing, as the accuracy may not be high enough to account for the myriad of variables at play. Moreover, it is to allow a design engineer to quickly make an estimate of how large the battery required will be to operate a device early in the design process, to allow weight and size requirements to be considered before a prototype is even produced, or fully revise the design if it appears unfeasible given other constraints.

## 6. Conclusions

In this paper, we have performed testing and analysed the results of several common low power LoRa prototyping boards. Further, we have performed in depth testing and analysis of the lowest power board in order to general a prediction model for the power required to operate under different use cases. The model produced has been tested experimentally and verified as accurate. We have performed some initial investigations into the effects of different common off-the-shelf sensors, which would could be employed with the system. Finally, a design method is presented enabling one to predict the power required to operate a battery- or energy harvesting-powered system and estimate the transmission interval ahead of time.

There are many design use cases in which physical constraints on the size or weight of the wireless sensor system is important. With the methods and data presented in this paper it is possible to estimate how much energy will be required to power the system by combining the model-generated energy estimate with simple calculations of duration and transmission interval to be able to assess if the proposed system is feasible or will require a higher energy input. This can reduce prototype iterations required or aid in the decision to abandon the proposed system based on hard requirements which cannot be changed.

## 7. Future Work

The model presented is able to estimate the power requirements for the LoRa unit based on various message sizes and settings. The impact that modifying these settings has on the signal strength and distance the unit is able to transmit is not known. LoRa has a theoretical maximum transmission distance of 20 km [9]. Further experiments could be performed in order to ascertain the transmission distance of LoRa boards using a combination of the settings discussed in this paper. Urban and rural testing could indicate likely thresholds for transmission settings, which using the model presented, could provide even more accurate prototype evaluation for wireless sensors.

## Figures and Tables

**Table 1:** Comparison of key wireless technology features. Adapted From: Security In Internet Of Things [11].

	Bluetooth LE	ZigBee	WiFi	Wi-Max	LoRa	LTE	Z-Wave
Standards	IEEE 802.15.1	IEEE 802.15.4	IEEE 802.11 ah	IEEE 802.16	IEEE 802.15 g	3GPP	Z-Wave Alliance
Network Types	P2P	Mesh	WLAN	MAN	LPWAN	GERAN	Mesh
Power Consumption	Very Low	Low	High	Medium	Very Low	Medium	Very Low
Data Rate	1 Mbps	0.25 Mbps	Up to 7000 Mbps	70 Mbps	250 kbps	0.1–1 Gbps	0.1 Mbps
Range	35 m	10–100 m	1 Km	50 Km	100 Km	28 Km/10 Km	30 m
Spectrum	2.4 GHz	2.4 GHz	2.4-5 GHz	2–11 GHz	868–915 MHz	700–2600 MHz	908.42 MHz

**Table 2:** Boards Chosen For Testing.

Board Name	Voltage	Supplier	Cost
LoRa-E5 Mini	5 V	Seed Studio	$19.90
LoRa-E5	5 V	Seed Studio	$24.90
Grasshopper	5 V	Tindie	$10.00
LOPY4	5 V	Pycom	$44.79

**Table 3:** Default Board Settings For Testing.

Parameter	Fixed Setting
Frequency Plan	863–870 MHz
Number of channels	16
Max EIRP	+16 dBm
Default data rate	DR0
Supply Voltage	5 V
Max Payload Size	51 bytes
Dwell time limitation	No
Default Channels	0, 1, 2

**Table 4:** Keithley 2450 SourceMeter Specifications [14].

Feature	Value
Basic Measurement Accuracy	0.012%
Measurement Range Selected	100 mA
Source Resolution	5 μA
Measure Resolution	100 nA
Noise	100 nA
Voltage Burden	<100 μV
Source Resolution Accuracy	0.025% + 15 μA
Temperature Coefficient	±0.15∗accuracy/C

**Table 6:** Casals et al. LoRaWAN Transmission States [15].

State Number	State Name
1	Wake Up
2	Radio Preparation
3	Transmission
4	Wait First Window
5	Receive First Window
6	Wait Second Window
7	Receive Second Window
8	Radio Off
9	Post Processing
10	Turn Off Sequence
11	Sleep

**Table 7:** Sensors tested for power consumption.

Sensor Type	Voltage	Chip/Code
Moisture Sensor	5 V	N/A
Thermocouple	5 V	DS18B20
Ultrasonic Sensor	5 V	HC-SR04
IR Distance Sensor	5 V	LM393

## Data Availability

All data used in the analysis presented in this paper is publicly. Available online: https://github.com/solomonould/lora_power_testing (accessed on 5 November 2021).

## References

[1] Raza U., Kulkarni P., Sooriyabandara M. (2017). Low Power Wide Area Networks: An Overview. IEEE Commun. Surv. Tutor..

[2] Augustin A., Yi J., Clausen T., Townsley W.M. (2016). A Study of LoRa: Long Range Low Power Networks for the Internet of Things. Sensors.

[3] Adelantado F., Vilajosana X., Tuset-Peiro P., Martinez B., Melià-Seguí J., Watteyne T. (2017). Understanding the limits of LoRaWAN. IEEE Commun. Mag..

[4] Vangelista L., Centenaro M. (2019). Worldwide Connectivity for the Internet of Things Through LoRaWAN. Future Internet.

[5] Bao L., Wei L., Jiang C., Miao W., Guo B., Li W., Cheng X., Liu R., Zou J. (2018). Coverage Analysis on NB-IoT and LoRa in Power Wireless Private Network. Procedia Comput. Sci..

[6] Petäjäjärvi J., Mikhaylov K., Pettissalo M., Janhunen J., Iinatti J. (2017). Performance of a low-power wide-area network based on LoRa technology: Doppler robustness, scalability, and coverage. Int. J. Distrib. Sens. Netw..

[7] Kiki M.J.M., Iddi I. (2021). Improved LORA Modulation Output in LEO Satellite Internet of Things. J. Electr. Eng. Technol..

[8] Mumtaz S., Alsohaily A., Pang Z., Rayes A., Tsang K.F., Rodriguez J. (2017). Massive Internet of Things for Industrial Applications: Addressing Wireless IIoT Connectivity Challenges and Ecosystem Fragmentation. IEEE Ind. Electron. Mag..

[9] Mekki K., Bajic E., Chaxel F., Meyer F. (2019). A comparative study of LPWAN technologies for large-scale IoT deployment. ICT Express.

[10] Singh R.K., Berkvens R., Weyn M. (2020). Synchronization and efficient channel hopping for power efficiency in LoRa networks: A comprehensive study. Internet Things.

[11] Aldowah H., Rehman S., Umar I. (2019). Security in Internet of Things: Issues, Challenges, and Solutions. arXiv.

[12] Liang R., Zhao L., Wang P. (2020). Performance Evaluations of LoRa Wireless Communication in Building Environments. Sensors.

[13] Pycom (2020). LoPy4 Datasheet. https://docs.pycom.io/datasheets/development/lopy4/.

[14] Tektronix (2021). Keithley 2460 SourceMeter Datasheet. https://www.tek.com/datasheet/2460-source-measure-unit.

[15] Casals L., Mir B., Vidal R., Gomez C. (2017). Modeling the Energy Performance of LoRaWAN. Sensors.

[16] Bäumker E., Garcia A.M., Woias P. (2019). Minimizing Power Consumption of LoRa^®^ and LoRaWAN for Low-Power Wireless Sensor Nodes. J. Phys. Conf. Ser..

[17] Kokten E., Çalışkan B., Karamzadeh S., Gelal E. (2020). Low-Power Agriculture IoT System with LoRa: Open Field Storage Observation. Electr. Control. Commun. Eng..

[18] Lopez Chalacan V.H. (2020). Performance Evaluation of Long Range (LoRa) Wireless Rf Technology for the Internet of Things (IoT) Using Dragino LoRa at 915 Mhz. Ph.D. Thesis.

[19] Gloria A., Dionisio C., Simões G., Sebastião P. LoRa Transmission Power Self Con?guration for Low Power End Devices. Proceedings of the 2019 22nd International Symposium on Wireless Personal Multimedia Communications (WPMC).

